# Role of Aquaporin 1 Signalling in Cancer Development and Progression

**DOI:** 10.3390/ijms18020299

**Published:** 2017-01-29

**Authors:** Yoko Tomita, Hilary Dorward, Andrea J. Yool, Eric Smith, Amanda R. Townsend, Timothy J. Price, Jennifer E. Hardingham

**Affiliations:** 1 Molecular Oncology, Basil Hetzel Institute, The Queen Elizabeth Hospital & Discipline of Physiology, School of Medicine, University of Adelaide, Adelaide, SA 5000, Australia; Jenny.Hardingham@sa.gov.au; 2 Molecular Oncology, Basil Hetzel Institute, The Queen Elizabeth Hospital, Woodville South, SA 5011, Australia; hilary.dorward07@gmail.com (H.D.); Eric.Smith@adelaide.edu.au (E.S.); 3 Discipline of Physiology, School of Medicine, University of Adelaide, Adelaide, SA 5000, Australia; Andrea.Yool@adelaide.edu.au; 4 Medical Oncology, The Queen Elizabeth Hospital & School of Medicine, University of Adelaide, Adelaide, SA 5000, Australia; Amanda.Townsend@sa.gov.au (A.R.T.); Timothy.Price@sa.gov.au (T.J.P.)

**Keywords:** aquaporin 1, carcinogenesis, tumour angiogenesis, tumour cell migration

## Abstract

Cancer is a major health burden worldwide. Despite the advances in our understanding of its pathogenesis and continued improvement in cancer management and outcomes, there remains a strong clinical demand for more accurate and reliable biomarkers of metastatic progression and novel therapeutic targets to abrogate angiogenesis and tumour progression. Aquaporin 1 (AQP1) is a small hydrophobic integral transmembrane protein with a predominant role in trans-cellular water transport. Recently, over-expression of AQP1 has been associated with many types of cancer as a distinctive clinical prognostic factor. This has prompted researchers to evaluate the link between AQP1 and cancer biological functions. Available literature implicates the role of AQP1 in tumour cell migration, invasion and angiogenesis. This article reviews the current understanding of AQP1-facilitated tumour development and progression with a focus on regulatory mechanisms and downstream signalling pathways.

## 1. Introduction

Cancer is a leading cause of mortality and morbidity, with 8.2 million cancer-related deaths estimated to have occurred world-wide in 2012 [1]. In the United States, the age-adjusted mortality rate from all cancers combined declined by an average of 1.5% per year between 2003 and 2012 [2]. A similar trend has been seen in Australia where the age-standardised mortality rate was estimated to have decreased by 20% from 209 per 100,000 in 1982 to 168 per 100,000 in 2014 [3]. Owing to the advances in technology and understanding of cancer pathogenesis, its treatment has evolved rapidly in the last few decades, resulting in improved cancer survival. However, a large proportion of cancer patients continue to succumb to the condition despite optimal treatments; the five-year survival rate for the period of 2007–2011 was 67% [3].

Systemic therapy such as chemotherapy, biological therapy and hormone therapy have become the standard of care in management of many types of cancers and are utilised in neoadjuvant, adjuvant or palliative settings. Unfortunately, tumours frequently become refractory to the therapy, resulting in recurrence or progression of the disease. Moreover, toxicity associated with currently available systemic therapy is not uncommon, often interfering with treatment administration. This highlights the ongoing demand for new biomarkers as possible sources of novel therapeutic targets.

Aquaporins (AQPs) are a family of small hydrophobic integral transmembrane proteins with predominant roles in trans-cellular water transport in response to osmotic gradients [4,5,6]. They are distributed throughout human tissues, although the majority are located in epithelium, endothelium and specialised cells such as erythrocytes, astrocytes, adipocytes and skeletal muscles [7]. AQPs have various known physiological roles; urine concentration in kidney tubules, epithelial fluid secretion of saliva, cerebrospinal fluid and aqueous humor production, cell migration required for angiogenesis and wound healing, regulation of brain water homeostasis, neural signal transduction, skin moisturisation, cell proliferation in wound healing and fat metabolism [8].

## 2. Aquaporin 1

**Figure 1:**
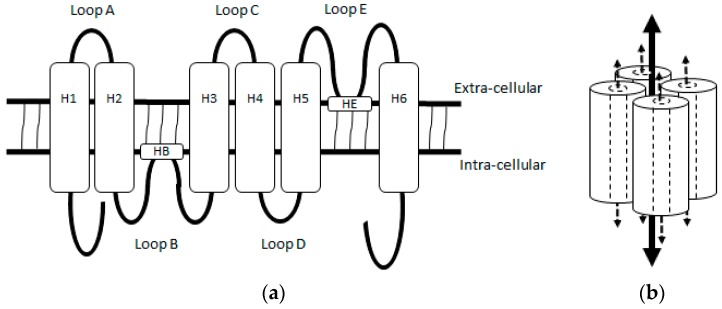
Structure of Aquaporin 1 (AQP1). (**a**) An illustration of aquaporin monomer showing 6 α-helical domains (H) 1–6 connected by 5 loops A-E. Loops B and E contain helical subunits (HB and HE) composed of a highly conserved NPA motif. When folded, helical subunits in Loop B and E bend internally, juxtaposing to form a water pore; (**b**) An illustration of aquaporin homotetramer. The dash arrows represent substrate paths through the water pores of the AQP1 monomers, and the solid arrow represents the proposed path for ion and gas through the central pore of homotetramer.

Aquaporin 1 (AQP1) was the first mammalian AQP reported being observed in erythrocytes and renal tubules, and was originally named channel-forming integral membrane protein of 28 kDa (CHIP28) [9]. Subsequent studies have demonstrated that it is physiologically distributed in the choroidal plexus, corneal endothelium, pain-processing C-fibres of the spinal cord and all vascular endothelial cells except in the central nervous system [8]. AQP1 is organised as a tetramer spanning the plasma membrane and the AQP1 monomer is a ~28 kDa protein consisting of six-tilted α-helical domains (H1–H6), connected by five loops (Loops A–E) (Figure 1). While a water pore formed by loops containing the two Asn-Pro-Ala (NPA) motifs located in Loop B and E transports water and solutes, a central pore of the AQP1 homotetramer transports gas and ions [10,11].

The primary function of AQP1 is water transport, as demonstrated using *Xenopus laevis* oocytes injected with cRNA for CHIP28 [12]. AQP1 additionally functions as a cyclic nucleotide-gated cation channel that is activated mainly by cGMP and indirectly by cAMP [13,14,15]. Yu and colleagues reported an interaction of cGMP with arginine-rich cytoplasmic Loop D, which facilitated its outward motion, leading to the hypothesis that cGMP-induced-conformational change in cytoplasmic Loop D mediates the gating of the central ion channel in AQP1 [16].

AQP1 is over-expressed in multiple human cancers including that of biliary duct, bladder, brain, breast, cervix, colon, lung, nasopharynx and prostate [17,18,19,20,21,22,23,24,25,26]. In the case of colon cancer, Moon and colleagues showed expression of AQP1 in colonic adenoma, primary and secondary colon cancer, but not in normal colonic mucosa, suggesting a role of AQP1 in the early stage of colon cancer tumorigenesis [19]. Additionally, its expression was reported to be associated with clinical characteristics known to be prognostic such as histological grade and status of lympho-vascular invasion and nodal involvement [18,22,23,27,28,29,30]. These findings suggested the link between AQP1 and cancer biological functions, which act to drive cancer development and progression. There has been close to a dozen of reviews published on the topic of AQPs and cancer and/or the therapeutic potential of AQP inhibitors in cancer treatment [5,6,31,32,33,34,35,36,37,38]. This, however, is the first review the authors are aware that is dedicated to discussion on the significance of AQP1 in cancer biology.

## 3. Proposed Mechanisms Underlying AQP1-Enhanced Tumour Progression

### 3.1. AQP1-Modulated Tumour Cell Migration and Invasion

The acquisition of a migratory and invasive phenotype by carcinoma cells is a crucial step in cancer progression, as it facilitates metastasis that accounts for 90% of human cancer death [39]. AQP1 plays a significant role in tumour cell migration and invasion. Hu and Verkman observed that AQP1 expression accelerated migration of mouse melanoma B16F10 and breast cancer 4T1 cell lines in vitro, with polarised expression of AQP1 observed at the leading edge of the migrating cells [40]. In mice, they observed that AQP1 expression increased cancer cell extravasation and lung metastases. These findings were supported by observations of Jiang who showed alteration of osmotic water permeability induced by over- or under-expression of AQP1 channels influenced the migratory property of HT20 human colon cancer cells in vitro; AQP1 over-expressing HT20 cells demonstrated increased extravasation in nude mice [41].

The importance of water fluxes across the plasma membrane in the facilitation of lamellipodia formation has been acknowledged in earlier reports on cell migration [42,43]. One proposed mechanism for AQP1-modulated tumour cell migration is induction of osmotic water flow across the plasma membrane by AQP1 in response to an osmotic gradient created by actin depolymerisation and active solute influx at the leading edge of migrating cells [44]. Water influx through AQP1 can increase hydrostatic pressure, causing local expansion of plasma membrane, followed by actin re-polymerisation to stabilise cell membrane protrusion. Chen and colleagues demonstrated infection of 603B cholangiocyte cells with *Cryptosporidium parvum*, an intracellular parasite, led to recruitment of host-cell AQP1 and SGLT1, a Na^+^/glucose co-transporter, to the attachment site with resultant localised water influx, and inhibition of AQP1 resulted in reduction in the parasite cellular invasion, which was dependent on host-cell membrane protrusion [45]. Alternative mechanism to explain APQ1 modulated tumour cell migration is changes in cell shape and volume of migrating cells during passage through tight environments and generation of hydrostatic forces induced by AQP1. Actin polymerization/depolymerisation and transmembrane ionic fluxes may facilitate osmotic water flow through AQP1 [46]. Stroka and colleagues also proposed an “Osmotic Engine Model”, involving an actin- and myosin-independent cell migration mechanism based on water permeation and active and passive ion transport through AQPs and Na^+^/H^+^ pumps for cell migration in confined microenvironments [47].

Migration of cells requires coordinated regulation of [Ca^2+^]_i_, intra- and extra-cellular pH, cellular membrane potential and volume, making ion channels and transporters integral components of cellular migration mechanism [48]. Cells establish polarity during directional migration and several ion channels and transporters have been suggested to polarise at the leading edge of migrating cells where they may play a role in triggering osmotic water flow across plasma membrane [49,50]. Ion channel and transporters such as AQPs, K^+^ channels, Na^+^/H^+^ exchanger and Na^+^/glucose co-transporter and calcium-regulatory transporter proteins are suggested to be involved in the key steps of tumour metastasis cascade, namely loss of cell-cell contacts, invasion of surrounding stroma and intra- and extra-vasation of vasculature [51,52]. Kourghi and colleagues showed several AQP1 ion channel blockers with no effect on water channel activity inhibited migration of HT29 cells and the magnitude of inhibition was dependent on the potency for AQP1 ion channel block [53]. Ion channel property of AQP1 alone may be sufficient to facilitate tumour cell migration in some cancers.

More recently, association of AQP1 with tumour microenvironment-driven tumour growth has been suggested. Pelagalli and colleagues demonstrated that treatment with bone marrow-derived mesenchymal stem cells (BM-MSCs) conditioned medium increased AQP1 expression with resultant enhanced migration and invasion of U2OS osteosarcoma and SNU-398 hepatocellular carcinoma cells, which were hampered by AQP1 inhibitor, tetraethylammonium chloride [54]. BM-MSCs have been shown to differentiate into cancer-associated fibroblasts which promote tumour progression and metastasis [55,56]. AQP1 may play an important role in the cross-talk between tumour microenvironment and tumour cells together with various growth factors, cytokines and chemokines.

### 3.2. AQP1-Modulated Tumour Angiogenesis

Tumour angiogenesis—the formation of new blood vessels within the tumour—is another fundamental property of cancer and is vital for tumour metastasis [57]. These vessels provide essential nutrients to sustain tumour growth, and a route by which the cancer cells can exit the tumour and enter the circulation. There has been an increasing recognition that ion channels and transports are concerned with neovascularization through mediating activation, proliferation, migration and differentiation of endothelial cells. Ion channels and transporters may serve as enzymes, chemical and mechanical sensors, receptors and scaffolding proteins [58]. Up-regulation of AQP1 was shown in the perivascular area of astrocytoma where infiltration of tumour cells occur, in contrast to the scarce expression in the necrotic centre, suggesting a link between AQP1 and tumour angiogenesis [22].

Saadoun and colleagues observed reduced density of tumour microvessels in AQP1 null mice subcutaneously implanted with melanoma cells, which delayed tumour growth, and prolonged the survival of the mice [59]. Abnormal tumour microvascular anatomy with reduced density resulting from inhibition of AQP1 expression was also reported by Nicchia and colleagues who tested RNA interference knockdown using AQP1 siRNA on mice subcutaneously implanted with B16F10 mouse melanoma cells, and by Esteva-Font and colleagues who examined the impact of AQP1 deficiency in mouse mammary tumour virus-driven polyoma virus middle T oncogene (MMTV-PyVT) mice which spontaneously developed epithelial cancer [60,61]. These findings were accompanied by reduced staining for vascular endothelial growth factor receptor 2 (VEGFR2), an indicator of angiogenic sprouting, and reduced expression of the endothelial marker, factor VIII [60,61].

Using endothelial cells isolated from AQP1 null mice, Saadoun and colleagues showed impaired migration of AQP1-deficient endothelial cells associated with abnormal vessel formation in vitro [59]. Silencing of AQP1 using siRNA in HMEC-1 human endothelial cells resulted in a lack of F-actin polarisation at the leading edge of the plasma membrane and failure of these cells to organise a cord-like network in vitro, the finding also demonstrated in AQP1-silenced WM115 human melanoma cells [62]. This implied that AQP1 facilitated the migration not only of tumour cells but also endothelial cells, enabling tumour angiogenesis. As endothelial cell migration is linked to vascular permeability, Clapp and Escalera proposed that enhanced vessel permeability facilitated by AQP1, which increases cellular water transport, initiates angiogenic cascade by inducing extravasation of plasma proteins needed as a scaffold for migrating endothelial cells [63].

### 3.3. AQP1-Modulated Tumour Proliferation

In comparison to tumour cell migration and tumour angiogenesis, the evidence for AQP1-facilitated tumour cell proliferation is less substantial. Inhibition of AQP1 activity in colon cancer cell line HT29 showed no effect on proliferation, while in another colon cancer cell line HCT-116, proliferation was reduced by 17% [64]. Mouse cancer cell lines, B16F10 and 4T1 with induced over-expression of AQP1 failed to show increased cell proliferation despite exhibiting increased extravasation and the formation of distant metastases [40]. Contrary to this, Hoque and colleagues observed enhanced cell proliferation in NIH-3T3 mouse embryo fibroblast cell line with forced AQP1 expression in vitro [20]. Similar finding was reported with rat pheochromocytoma cell line PC12 [65]. Down-regulation of AQP1 expression with shRNA in two osteosarcoma cell lines U2OS and MG63 was accompanied by inhibition of proliferation [66]. AQP1 inhibition by an inhibitor AqB050 or siRNA knockdown was reported to result in a reduction in cell proliferation in primary malignant mesothelioma cells harvested from pleural effusions [67]. 

Resistance to apoptosis has been proposed as a part of the mechanism underlying enhanced cell proliferation of AQP1 expressing cells [20,66]. In addition to reduced induction of apoptosis on nocodazole treatment to synchronise cell cycle, transfection of PC12 cells with AQP1 cDNA increased the proportion of cells in S and G2/M phases and this was associated with enhanced expression of cyclin D1 and E1, key proteins needed for cell cycle progression through G1 phase and G1/S transition, respectively [68]. Progression through the cell cycle is accompanied by increase in cell volume, while apoptosis involves reduction in cell volume [69,70]. With AQP1 over-expressing PC12 cells exhibiting a larger cell size and higher intracellular complexity compared to the wild-type control, Galan-Cobo and colleagues postulated that the cell morphology change induced by AQP1 over-expression facilitates tumour cell progression through the cell cycle and antagonizes apoptotic process [68].

## 4. Hypoxia-Facilitated Glycolysis as an Inducer of AQP1 Expression in Tumour Cells

**Figure 2:**
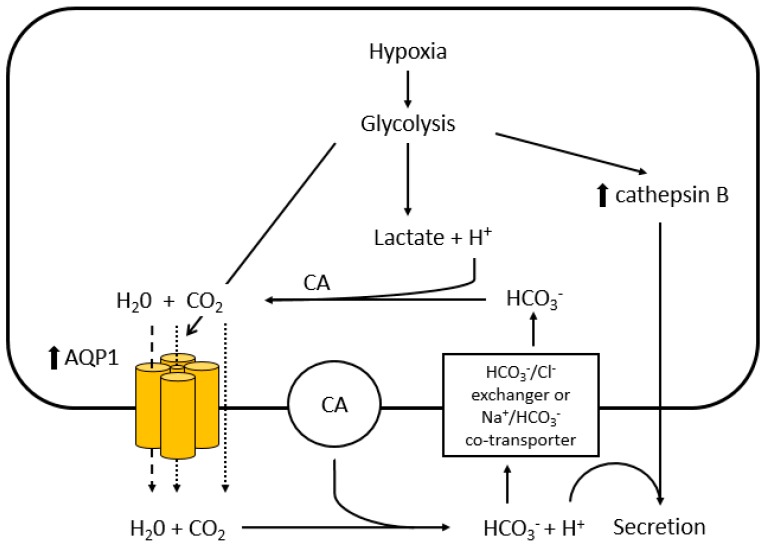
Putative mechanism of hypoxia-facilitated AQP1 expression in tumour cells. Hayashi and colleagues postulated that hypoxia facilitates AQP1 expression through glycolysis [72]. Hypoxia-induced glycolysis produces lactic acid and enhances the transcription of AQP1 and cathepsin B through E-box/ChoRE. The elevated lactic acid causes intracellular acidosis and an excess in H^+^. The excess H^+^ are converted to H_2_O and CO_2_ through the reaction with HCO_3_^−^ which is catalyzed by intracellular carbonic anhydrases (CA). The excess H_2_O generated exits the tumour cell through the water pores of the up-regulated AQP1 (dashed line - -) to prevent cytotoxic oedema, while CO_2_ may leave the cell through the central pore of the AQP1 tetramer or diffuse through the plasma membrane (dotted line ·····). The H_2_O released from the cell is recycled to regenerate H^+^ by membrane-bound CA with resultant acidification of the extracellular compartment, stimulating production and secretion of cathepsin B.

Owing to rapid cellular proliferation, hypoxia is a feature common to many types of cancer and it contributes to tumour progression and resistance to therapy [71]. Hayashi and colleagues observed enhanced AQP1 expression by hypoxia in 9L rat glioblastoma cells which correlated with the extent of glycolysis, and postulated that AQP1 expression is induced by hypoxia-facilitated glycolysis (Figure 2) [72]. In response to the intracellular lactic acidosis caused by hypoxia, tumour cells are required to shuttle H^+^ to the extracellular compartment; this may involve reaction of H^+^ and HCO_3_^−^ catalysed by the cytosolic carbonic anhydrases [73]. H_2_O produced as a result could be subsequently transported by AQP1 to the extracellular compartment to mitigate cytotoxic oedema. Hypoxia-facilitated AQP1 expression may in fact occur through the E-Box/ChoRE transcriptional element present in AQP1 gene promoter, which is known to increase gene transcription in response to increased glucose consumption and metabolism [74,75]. C-Myc, often up-regulated in tumour cells is known for its ability to directly stimulate the transcription of E-Box-containing genes thus up-regulating AQP1 expression [76].

The role of hypoxia in induction of AQP1 expression in tumour cells is further supported by Abreu-Rodrigues and colleagues who demonstrated that hypoxia-enhanced transcription of AQP1 through increased activation of the AQP1 promoter and identified putative hypoxia-inducible transcription factor (HIF) binding sites in the promoter region of murine AQP1 gene by bioinformatic analysis [77]. Presence of the consensus sequence for the HIF binding site in the promoter region of human retinal vascular endothelial cell (HRVEC) AQP1 gene has also been reported by Tanaka and colleagues and the promoter activity of HRVEC AQP1 was again increased under hypoxia [78]. Correlation of AQP1 and HIF1 expression in breast cancer tissues was demonstrated by Yin and colleagues [79]. Unlike the wild-type cells, AQP1 over-expressing clone of PC12 cells induced stable expression of HIF-2α under chronic hypoxia [65]. Abolishment of stimulatory effect of hypoxia on AQP1 expression by a mutation in hypoxia response element (HRE), which is known to bind HIF1, was shown in the PC-3M human prostate cancer cell line [80]. As mutations of the HIF binding sites did not abrogate the hypoxia response completely, other hypoxia-responsive transcription factors such as EGR1, SP1, ETS1, AP1, CREB1 and NFκB are considered to contribute to hypoxic activation of AQP1 [77].

Tie and colleagues additionally reported hypoxia-induced AQP1 expression was prevented by a P38 MAPK inhibitor in a dose-dependent manner, with PKC and intracellular calcium ion being responsible for the activation of the pathway [80]. All three MAPK pathways (ERK, P38 and JNK) have been shown to be involved in hypertonicity-induced AQP1 expression in mIMCD-3 mouse medullary cells and products of immediate early response gene such as Fos, Myc and Jun oncogenes may act as transcription factors downstream of MAPK pathways, facilitating hypoxia-induced AQP1 promotor activation [20,81].

Hypoxia also facilitates tumour angiogenesis. An experiment using a mouse endothelial cell line EOMA which over-expresses mutated forms of HIF-1α resistant to degradation confirmed direct participation of HIF-1α in the hypoxic up-regulation of AQP1 promoter [77]. Augmentation of cyclooxygenase-2-dependent prostaglandin E_2_ release with resultant up-regulation of VEGF and AQP1 was shown in human umbilical vein endothelial cells (HUVECs) and this was accompanied by enhanced cellular proliferation, migration and tube formation [82]. 

## 5. Regulation of AQP1 Activity

Cyclic nucleotide and protein kinase pathways are the two regulatory mechanisms currently proposed to be involved in the activation of AQP1 channel activity. Cyclic nucleotides such as cAMP are known for their role as second messengers in both hormone and ion-channel signalling in eukaryotic cells either directly or via activation of protein kinases and subsequent phosphorylation of substrate proteins [83]. Patil and colleagues demonstrated cAMP increased membrane permeability of water in *Xenopus oocytes* injected with AQP1 cRNA [84]. Under hyperosmolar conditions, AQP1 expression was augmented by the agonist of cAMP, vasopressin through increased translocation of AQP1 from cytosol to plasma membrane in mouse inner medullary collecting duct cells [85].

Han and Patil [86] found the catalytic subunit of cAMP-dependent protein kinase (PKA) significantly increased the amount of phosphorylated AQP1 protein. AQP1 lacks the typical PKA consensus sequence Arg-Arg-X-Ser/Thr for phosphorylation; however, several proteins that are phosphorylated by PKA at Arg-X-Ser sequence are known to exist and bovine AQP1 was previously shown to exhibit Arg-X-Ser sequence at Ser-238 [87,88,89]. 

Analysis of AQP1 amino acid sequence previously identified two consensus sites for protein kinase C (PKC) phosphorylation at Thr^157^ and Thr^239^, and pharmacological activation of PKC led to enhanced AQP1-dependent water permeability and AQP1 cationic currents in AQP1-expressing *Xenopus oocytes*, which were abolished by mutated AQP1 lacking both consensus PKC phosphorylation sites [90]. Increase in intracellular cyclic nucleotide levels in *Xenopus oocytes* expressing mutated AQP1 lacking both PKC phosphorylation sites still resulted in enhanced current, indicating the PKC pathway and cyclic nucleotide pathways independently regulated AQP1.

## 6. Downstream Effectors and Signalling Pathways in AQP1-Mediated Tumour Progression

Despite the extensive literature supporting the involvement of AQP1 in cancer development and progression, the exact signalling pathways involved are yet to be elucidated. Several molecules and intracellular pathways have been implicated in AQP1 downstream signalling.

### 6.1. β-Catenin and Lin-7

**Figure 3:**
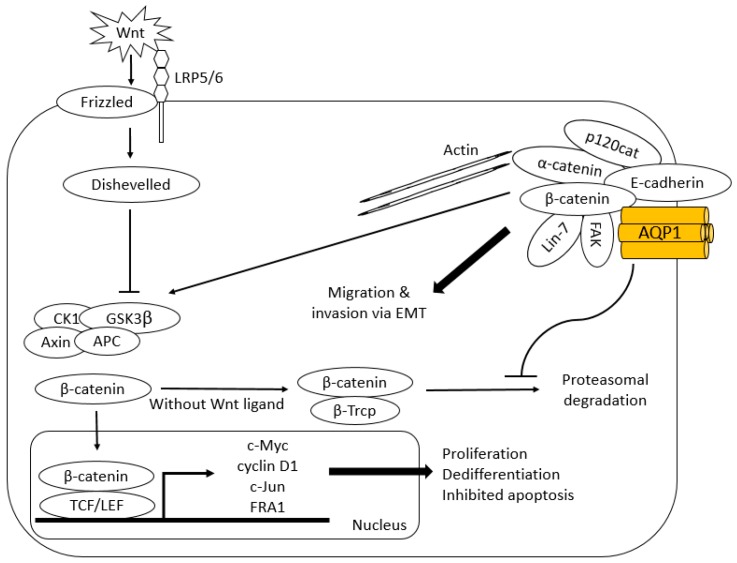
Proposed interaction of AQP1 with Wnt/ β-catenin signalling and cadherin-catenin complex. Interaction of AQP1 with β-catenin negates its proteasomal degradation augmenting Wnt signalling pathway. Once transported to nucleus, β-catenin serves as a co-activator for TCF to activate Wnt responsive genes including those responsible for tumorigenesis such as c-Myc, cyclin D1, c-Jun and FRA1. AQP1 also stabilises cadherin/β-catenin/Lin-7/F-actin complex to enhance migratory and invasive capacity of tumour cells. Given the co-immunoprecipitation with β-catenin, it is proposed FAK is a part of cadherin-catenin complex [91].

Meng and colleagues demonstrated co-immunoprecipitation of AQP1 and β-catenin with up-regulation of β-catenin when mesenchymal stem cells (MSC) were made to over-express AQP1 [91]. β-catenin functions as an intracellular signal transducer in canonical Wnt signalling pathway and regulates gene transcription (Figure 3) [92,93]. In the absence of Wnt ligand, cytoplasmic β-catenin forms a complex with Axin, APC, GSK3β and CK1 and becomes phosphorylated. This allows β-catenin to be recognized by E3 ubiquitin ligase β-Trcp for proteasomal degradation. In the presence of Wnt ligand, a receptor complex forms between Frizzled and LRP5/6 resulting in recruitment of Dishevelled, which phosphorylates LRP5/6 with subsequent Axin recruitment. This disrupts Axin-mediated phosphorylation/degradation of β-catenin, allowing β-catenin to transport to the nucleus where it serves as a co-activator for TCF to activate Wnt responsive genes including those responsible for tumorigenesis such as c-Myc, cyclin D1, c-Jun and FRA1.

The pathway is concerned with establishment and maintenance of stem cells and selective destruction of a cytosolic β-catenin pool resulted in a loss of tumorigenic potential in the mouse model of a colon cancer cell line DLD1 [94]. c-Myc, cyclin D1 and the components of AP-1 transcription factor complex, c-Jun and FRA1 have been proposed to be the transcriptional targets of the Wnt signalling pathway, and are relevant in human tumorigenesis in that they are known to control cell cycle progression (G1/S transition), as well as proliferation, differentiation and apoptosis [95,96,97]. Because treatment of AQP1-silenced WM115 human melanoma cells with the proteasome inhibitor MG132 induced the recovery of β-catenin, it has been suggested that AQP1 negates proteasomal degradation of β-catenin to augment Wnt signalling pathway [62]. Yun and colleagues demonstrated AQP1-mediated enhanced β-catenin expression in pulmonary arterial myocytes required AQP1 C-terminal tail, which contains protein binding sites [98]. Interaction of AQP1 with β-catenin via its C-terminus may hinder association of β-catenin with APC/Axin destruction complex for proteasomal degradation [93].

β-catenin also plays a role in cell-cell adhesion. Reorganisation of cadherin-catenin complexes is implicated in epithelial-mesenchymal transition (EMT), which enables epithelial tumours to become invasive [99]. Silencing of AQP1 using siRNA in WM115 human melanoma and HMEC-1 human endothelial cells resulted in a lack of polarisation of F-actin at the leading edge of plasma membrane and failure of these cells to organise a cord-like network in vitro [62]. Lin-7, another component of cadherin-catenin complex which interacts with PDZ domain of β-catenin, was shown to co-immunoprecipitate with AQP1 in WM115 human melanoma and HMEC-1 human endothelial cells and its protein expression was reduced by siRNA down-regulation of AQP1. Researchers have speculated AQP1 stabilises the cadherin/β-catenin/Lin-7/F-actin complex to enhance the migratory and invasive capacity of tumour cells [62].

Cadherin is currently considered to negatively regulate Wnt signalling by sequestering cytoplasmic β-catenin as it shares the binding site on β-catenin with adenomatosis polyposis coli protein of Wnt signalling; however, the exact interplay between the cytoplasmic pool of β-catenin for Wnt signalling pathway and cadherin-bound β-catenin at plasma membrane is poorly understood [100]. Further research into interaction of AQP1 with each pool of β-catenin is needed.

### 6.2. FAK

**Figure 4:**
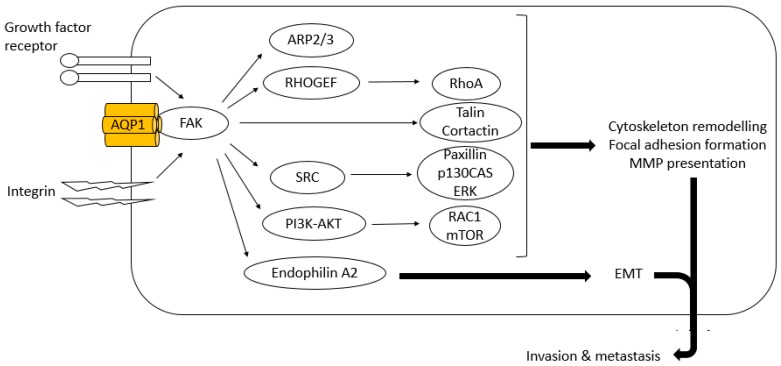
Suggested facilitation of FAK signalling by AQP1 to enhance tumour cell invasion and metastasis. FAK promotes tumour cells to gain invasive cell phenotypes through changes in cytoskeleton and focal adhesion dynamics, and expression of MMPs and epithelial-mesenchymal transition (EMT) markers. FAK induces cytoskeletal rearrangement through its interaction with ARP2/3 and FAK-associated proteins such as talin and cortactin [101]. FAK also stimulates formation, maturation and turnover of focal adhesions by activating paxillin and Rho family of GTPases, RhoA and Rac1. Increase in cell surface presentation of MMPs occurs through enhanced activities of SRC-mediated p130CAS and PI3K-AKT-mTOR signalling cascade. Association of FAK with endophilin A2 induces transcription of EMT markers. AQP1 is proposed to stabilize FAK augmenting its pro-tumour cell invasion and metastasis properties.

FAK is a cytoplasmic tyrosine kinase that induces growth factor receptor- and integrin-mediated signal transduction. It is implicated in tumour progression; activated and/or over-expressed FAK is found in a variety of human cancers. FAK contributes to tumour progression by enhancing tumour cell motility and EMT and by promoting tumour vascularisation (Figure 4) [101]. Vascular endothelial growth factor-A (VEGF-A)/VEGF or angiopoietin-1 signalling activates PI3K/AKT through the action of FAK to promote migration, sprouting and angiogenesis of endothelial cells [102]. It is postulated AQP1 partly exhibits its tumorigenic effect through activation of FAK signalling. FAK co-immunoprecipitated with AQP1 as well as β-catenin in AQP1 over-expressing MSC and depletion of FAK abolished AQP1-driven MSC migration (Figure 3) [91]. As over-expression of AQP1 did not alter its expression at mRNA level, the authors concluded that up-regulation of FAK seen in AQP1 over-expressing MSCs again occurred at post-translation level.

### 6.3. MMP2 and MMP9

Expression levels of MMP2 and MMP9 were found to be down-regulated by AQP1 siRNA in LTEP-A2 and LLC lung cancer cell lines [103]. These enzymes degrade type IV collagen, the major structural component of basement membrane and activate TGF-β to promote EMT [104]. Up-regulation of MMPs are common in many tumour cells and has been shown to facilitate tumour cell migration in vitro and metastasis in vivo [105,106,107,108]. FAK was reported to induce secretion of MMP2 and MMP9 in mouse fibroblasts in a Concanavalin-dependent manner [109]. Wnt signalling has been reported to induce MMP2 and MMP9 expression in effector T cells [110]. It is speculated AQP1 enhances the activity of MMP2 and MMP9 through FAK and Wnt signalling pathways, augmenting migration of tumour cells and endothelial cells. 

### 6.4. RhoA and Rac

Rho GTPases are involved in cell cytoskeleton rearrangements, migration and proliferation, and transformation. Aberrant regulation of Rho GTPases has been implicated in tumour progression via several mechanisms; loss of cellular polarity, stimulation of dedifferentiation, alteration of cell-cell and cell-matrix adhesion which allows tumour cells to become invasive and metastasise to distant sites via vascularization of tumours and intravasation of tumour cells [111]. RhoA and Rac1, in particular, have been shown to regulate mesenchymal and amoeboid movements, respectively, where RhoA drives actomyosin contraction, while Rac1 mediates cell polarisation and lamellipodia formation [112]. These two modes of cell motility play an important role in tumour cell invasion in three-dimensional extracellular matrix.

The association of AQP1 with RhoA and Rac has been previously suggested by Jiang who observed increase in the activation of these small G proteins in migrating HT20 colon cancer cells over-expressing AQP1, with more frequent polarised expression of actin at the cells’ leading edges. Impaired expression of RhoA was also seen in U2OS and MG63 cells following AQP1 down-regulation by shRNA [66]. Both RhoA and Rac1 are known to be involved in FAK-mediated tumour invasion and metastasis via Rho guanine exchange factor and the PI3K signalling pathway (Figure 4), and this may be one of the mechanisms whereby AQP1 influences the activities of Rho GTPases [101].

### 6.5. Cathepsin B

As described earlier, hypoxia-induced glycolysis may enhance AQP1 expression in tumour cells to maintain their viability. This is thought to cause acidification of the extracellular compartment with increase in secretion of a lysosomal cysteine protease cathepsin B (Figure 2) [113]. Up-regulation of cathepsin B together with AQP1 and LDH in 9L cells under glycolytic conditions was previously demonstrated [72]. High levels of expression of cathepsin B have been observed in epithelial tumours and glioblastoma, and are implicated in tumour development and progression via initiation, proliferation, angiogenesis, invasion, inflammation, apoptosis and metastasis [114]. Cathepsin B has been suggested to be a part of proteolytic network that involves MMPs and the plasminogen activator cascade, which may contribute to enhanced motility, invasion and angiogenesis [115,116,117]. Cathepsin B has additionally been thought to cleave anti-apoptotic proteins, such as Bcl-2, Bcl-xl and Bak, resulting in deregulation of tumour cell apoptosis [118].

### 6.6. TGF-β

Whether TGF-β plays any role in AQP1-facilitated tumour progression remains to be further evaluated. There have been two articles published in which expression of TGF-β was examined in AQP1 down-regulated tumour cells. Gene set enrichment analysis performed by Wu and colleagues indicated a positive correlation between AQP1 over-expression and TGF-β signalling pathway in osteosarcoma and expression of TGF-β1 and TGF-β2 was reduced in U2OP and MG63 cell lines when their AQP1 expression was down-regulated by shRNA [66]. Conversely, no alteration in the level of TGF-β was observed in AQP1 siRNA-treated LTEP-A2 and LLC cells [103]. The conflicting results reported may be secondary to the paradoxical action of TGF-β during tumour progression; while it initially supresses tumour development by regulation of cell proliferation, differentiation, adhesion and tumour microenvironment, TGF-β can manifest its pro-tumorigenicity by inducing uncontrolled cell proliferation, loss of apoptosis, EMT, sustained angiogenesis and evasion of immune surveillance [119].

## 7. Perspectives

AQP1 is a promising diagnostic tool and a plausible therapeutic target. Heavy metal ions and organic small molecules have been recognised as inhibitors of AQP1. A derivative of the loop diuretic bumetanide known as AqB013 blocks the water channel function of AQP1 and AQP4 [34]. Our group previously demonstrated that AqB013 significantly reduces both migration and invasion properties of HT29 colon cancer cells which endogenously express AQP1, and suppresses endothelial tube formation of HUVECs in vitro [64]. In the management of colorectal cancer (CRC), adjuvant chemotherapy is routinely offered to Stage III patients; however, the indication for such treatment in Stage II disease is less certain as the risk of toxicity from chemotherapy is likely to outweigh its benefits [120]. On the other side of the disease spectrum, chemotherapy and biological therapy against epidermal growth factor receptor (EGFR) and vascular endothelial growth factor (VEGF) are the primary treatments for advanced diseases; however, cancer cells commonly become refractory to these agents. New therapeutic agents are being sought to improve the outcomes of CRC. AQP1 inhibitors have potential clinical utility. For patients with localised disease, AQP1 inhibitors may reduce recurrence without the undue toxicities of currently administered adjuvant chemotherapy. Bumetanide has long been used to treat oedema and the incidence of clinically significant side-effects is very low. In advanced disease, given their expected anti-angiogenic property, AQP1 inhibitors may be combined with anti-VEGF agents, if not substitute for them following the development of resistance. Clinical utility of AQP1 inhibitors may apply to other types of cancer that are known to over-express this transmembrane protein.

## 8. Conclusions

AQP1 is an integral transmembrane protein with dual water and ion transport functions. Growing evidence supports its involvement in the development and progression of many types of cancer through AQP1-facilitated tumour cell migration, invasion, angiogenesis and possibly through tumour cell proliferation. Although the exact regulatory mechanisms and the actions of AQP1 on intracellular signalling pathways are yet to be fully uncovered, hypoxia appears to be one of the inducers of AQP1 over-expression in tumour cells, in combination with important downstream effectors including β-catenin, FAK and the Rho family of GTPases known for their role in tumorigenesis.

AQP1 is a strong cancer biomarker candidate and further research to elucidate the tumorigenic properties of AQP1 is awaited. Establishment of AQP1 as a cancer biomarker potentially encourages screening and stratification of susceptible patients for early cancer diagnosis, prognostication of cancer patients and prediction of treatment efficacy. Furthermore, AQP1 is a plausible novel therapeutic target and may enable development of effective cancer therapeutics.

## Abbreviations

AQP1	Aquaporin 1
AQPs	Aquaporins
BM-MSCs	Bone marrow-derived mesenchymal stem cells
CHIP28	Channel forming integral membrane protein of 28 kDa
CRC	Colorectal cancer
EGFR	Epidermal growth factor receptor
EMT	Epithelial-mesenchymal transition
HIF	Hypoxia-inducible transcription factor
HRE	Hypoxia response element
HRVEC	Human retinal vascular endothelial cell
HUVECs	Human umbilical vein endothelial cells
MMTV-PyVT	Mouse mammary tumor virus-driven polyoma virus middle T oncogene
MSC	Mesenchymal stem cells
NPA	Asparagine-proline-alanine
PKA	cAMP-dependent protein kinase
PKC	Protein kinase C
VEGF	Vascular endothelial growth factor
VEGF-A	Vascular endothelial growth factor-A
VEGFR2	Vascular endothelial growth factor receptor 2
